# Brain activity dynamics in human parietal regions during spontaneous switches in bistable perception

**DOI:** 10.1016/j.neuroimage.2014.12.018

**Published:** 2015-02-15

**Authors:** Fukuda Megumi, Bahador Bahrami, Ryota Kanai, Geraint Rees

**Affiliations:** a Institute of Cognitive Neuroscience, University College London, 17 Queen Square, London WC1N 3AR, United Kingdom; b Sackler Center for Consciousness Science, School of Psychology, University of Sussex, Pevensey 1, Brighton BN1 9QH, United Kingdom; c Wellcome Trust Centre for Neuroimaging, University College London, 12 Queen Square, London WC1N 3BG, United Kingdom

## Abstract

The neural mechanisms underlying conscious visual perception have been extensively investigated using bistable perception paradigms. Previous functional magnetic resonance imaging (fMRI) and transcranial magnetic stimulation (TMS) studies suggest that the right anterior superior parietal (r-aSPL) and the right posterior superior parietal lobule (r-pSPL) have opposite roles in triggering perceptual reversals. It has been proposed that these two areas are part of a hierarchical network whose dynamics determine perceptual switches. However, how these two parietal regions interact with each other and with the rest of the brain during bistable perception is not known. Here, we investigated such a model by recording brain activity using fMRI while participants viewed a bistable structure-from-motion stimulus. Using dynamic causal modeling (DCM), we found that resolving such perceptual ambiguity was specifically associated with reciprocal interactions between these parietal regions and V5/MT. Strikingly, the strength of bottom-up coupling between V5/MT to r-pSPL and from r-pSPL to r-aSPL predicted individual mean dominance duration. Our findings are consistent with a hierarchical predictive coding model of parietal involvement in bistable perception and suggest that visual information processing underlying spontaneous perceptual switches can be described as changes in connectivity strength between parietal and visual cortical regions.

## Highlights

• Two parietal regions involve spontaneous perceptual switches.
• The two parietal regions and V5/MT form hierarchical model.
• Strength of DCM parameters predicts individual switch frequency.

## Introduction

The human visual system processes complex and ambiguous visual information leading to subjective perceptual experience. The underlying neural mechanisms have been extensively investigated using bistable perception stimuli such as the Necker cube and binocular rivalry. Such stimuli induce different and spontaneously varying percepts while visual information projected on the retina remains unchanged. Functional magnetic resonance imaging (fMRI) studies suggest that human fronto-parietal brain regions may play a critical role in resolving such ambiguity in visual information and forming a unitary conscious percept (Kleinschmidt et al., 1998; Lumer et al., 1998; Sterzer and Kleinschmidt, 2007). In addition, transcranial magnetic stimulation (TMS) of the human parietal cortex demonstrates the causal involvement of distinct parietal regions in perceptual changes during bistable perception (Carmel et al., 2010; Kanai et al., 2010, 2011; Zaretskaya et al., 2010). Specifically, stimulation of the right anterior superior parietal (r-aSPL) and the right posterior superior parietal lobule (r-pSPL) has led to opposite effects on perceptual reversals (Kanai et al., 2011) leading to the suggestion that these two areas may be parts of a hierarchical network whose dynamics play a causal role in perceptual switches in bistable perception.

**Fig. 1:**
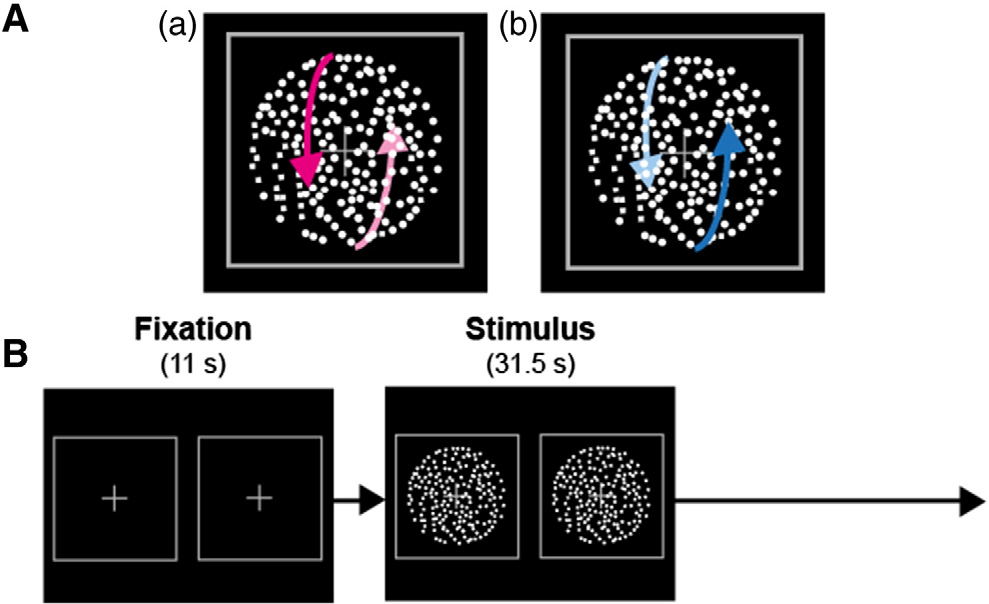
Illustration of the stimuli and the experimental procedure. (A) Our structure-from-motion (SFM) stimuli, which consisted of moving white dots, typically cause two exclusive alternating percepts: a sphere rotating either toward (a) or away (b) from the viewer. Note that the size of the white dots is magnified in this figure for visualization. (B) In fMRI session, SFM stimuli were presented on the screen for 30.5 s (15 EPI volumes). Participants were asked to report their percept by pressing or holding one of three buttons (toward, away, or not sure/mixture) during stimulus presentation. Note that stimuli were presented dichoptically in order to add disparity information for the replay condition: the participants used prism glasses and the screen was split by a black cardboard divider to aid fusion and ensures monocular presentation of each image.

To test this hypothesis, we used functional magnetic resonance imaging (fMRI) to record brain activation, while participants viewed a structure-from-motion stimulus (see Fig. 1), which leads to spontaneous alternations between two exclusive perceptual states (sphere rotating toward or away from the viewer). We applied dynamic causal modeling (DCM) analysis to test a specific model of connectivity proposed previously (Kanai et al., 2011). The advantage of using DCM is that we can express changes in brain dynamics associated with an experimental condition and directly compare the quantitative agreement between competing models and empirically observed Blood Oxygenation Level Dependent (BOLD) dynamics.

Based on a previous study (Kanai et al., 2011), we hypothesized that the anterior and posterior subregions of the right superior parietal lobule (r-aSPL and r-pSPL, respectively) and the motion sensitive visual area V5/MT form a hierarchical network structure with area V5/MT at the bottom and r-aSPL at the top level of the hierarchy. We predicted that reciprocal connections between them should mediate their dynamical interactions during perceptual rivalry and that the strength of the dynamical modulations of these connections should correlate with individual differences observed in participants' behavior in bistable perception. We functionally identified the three regions of interest (r-aSPL, r-pSPL, and, and right V5/MT, r-V5) using standard fMRI analysis approaches and then asked which DCM model structure and dynamics best explained information flow among these three regions and whether brain dynamics represented as parameters in the DCM model predicted inter-individual variance in percept dominance duration.

## Method

### Participants

Eighteen healthy participants (10 females, right-handed, ages 18 to 39, mean age ± standard deviation, SD: 26.0 ± 6.2 years) participated in this study. All participants had normal or corrected-to-normal vision with contact lenses. We obtained written informed consent from all participants. The local ethics committee approved the experiments.

### Experimental design

#### General procedure

We used structure-from-motion (SFM) stimuli (see Fig. 1) and recorded reports of spontaneous fluctuations in bistable perception (rivalry condition) and stimulus-driven changes (replay condition). Participants were instructed to look at the screen through prism glasses (Schurger, 2009) and report their subjective percept (the direction of rotation of the sphere) by holding one of three buttons; one for each of the two rotation directions, and one for mixture of two percepts or when the direction of rotation was unclear).

Prior to the fMRI session, participants underwent short behavioral testing outside the scanner to ensure that they could achieve stereopsis with our experimental setup and their reported percept durations were in the suitable range (3 to 10 s) for the fMRI experiment.

#### Apparatus

Stimuli were presented on the screen mounted on the MRI head coil using a JVC DLA-SX21 projector. Participants viewed the screen (the screen size was 27 cm × 21 cm; spatial resolution was 1024 × 768) through a mirror attached to the MRI coil. A viewing distance was approximately 72 cm. For dichoptic stimulus presentation, participants used prism glasses (lenses with 4 prism dioptres base out) and a black cardboard partition was attached to the head coil to divide the screen and the mirror into two areas for separate presentations to the left and right eye.

#### Stimuli

For the rivalry condition, a vertically spinning sphere (3.1° diameter) comprising 200 full-contrast white dots was presented to each eye for a structure from motion task (Kanai et al., 2010). Spheres were created using PsychToolbox 3 under MATLAB (The Mathworks, Inc.) and they were presented against a black background. The white dots moved sinusoidally upwards and downwards at an angular velocity of 120 degree/s. A fixation cross (0.1° in height and width) was superimposed at the center of each sphere. The spheres were surrounded by a square frame to help participants to maintain stable vergence and were presented at the same position relative to the fixation points to ensure that the direction of spin was ambiguous in the rivalry condition.

For the replay condition, binocular disparity was computed for each dot so that stimuli were embedded with unambiguous disparity cues and participants could perceive stereoscopic depth without difficulty. Fixation points, the spheres, and squares were aligned to the center of the illusory 3D spheres. Unlike binocular rivalry (Knapen et al., 2011) or the Lissajous figure (Weilnhammer et al., 2013), SFM typically does not induce a high proportion of mixed percepts. We confirmed for our stimulus configuration that the total duration of mixture of two alternative percepts was indeed very short (2.23% of the total duration of stimulus presentation per MRI run). We therefore focused on perceptual switches between two alternative percepts for the analysis.

#### Experimental procedure

On each trial, the ambiguous rotating sphere was presented continuously for 31.5 s (15 EPI volumes) followed by a fixation period (11 s, 5 EPI volumes).

Each MRI run consisted of 10 trials (five trials for rivalry condition and replay condition respectively), started with rivalry trial, and the order of rivalry and replay trials was pseudo-randomized. In a subset of the subsequent trials of the same run, the percept reported during the rivalry condition was replayed. The order of rivalry and replay trials was randomized across runs and participants. Participants performed the task for 4 to 7 runs in the MRI scanner (mean ± SD: 6.4 ± 0.9). Mean dominance duration during rivalry condition and replay rate (percentage of correct button response to the disambiguated sphere's direction of rotation on the screen, judged at each screen frame) was computed from MRI-compatible button response.

### MRI data acquisition

Images were obtained using a 3T Siemens Magnetom Trio MRI at the Wellcome Trust Centre for Neuroimaging at University College London. MRI data were collected with the fitted 32-channel head coil. Blood Oxygen Level Dependent (BOLD) signals were measured using an echo planar imaging (EPI) sequence (volume repetition time, 2.1 s; echo time, 30 ms; flip angle, 90°). EPI image contained 30 axial slices (3 mm thickness, ascending slice order), voxel size was 3 mm × 3 mm × 3 mm, and the field of view was 192 mm × 192 mm. T1-weighted structural images were acquired with 1 × 1 × 1 mm. Phase image and magnitude images were also obtained to compute a fieldmap (Hutton et al., 2002).

### MRI data processing

Statistical Parametric Mapping software (SPM8 and SPM12: Wellcome Trust Centre for Neuroimaging, http://www.fil.ion.ucl.ac.uk/spm) was used to process MRI data. The first five EPI volumes were discarded to allow for T1 equilibration. For preprocessing the EPI images, first, the EPI images were then realigned and unwrapped based on fieldmap images using the FieldMap toolbox in SPM8 (Hutton et al., 2002). EPI images were spatially normalized to the Montreal Neurological Institute (MNI) stereotactic template. Data were smoothed spatially with a Gaussian kernel of 8 mm full-width at half-maximum.

Statistical parametric mapping analysis was performed using the general linear model (GLM) approach. As a first step, GLM parameters were computed at the individual-level (fixed effects). The design matrix of the model contained four regressors: fixation, visual stimulation, spontaneous perceptual switch (rivalry-switch), and stimulus-driven perceptual switch (replay-switch). Visual stimulation and fixation periods were modeled using a box-car function, which represented the onset and duration of stimulus presentation and fixation period. Rivalry-switch and replay-switch were modeled with an impulse function. All regressors were then convolved with a canonical hemodynamic response function implemented in SPM8. In order to estimate actual timing of switch events, reaction time to press a button (mean reaction time across participants ± SD: 0.90 ± 0.43 s) was computed as the interval between replay stimulus change and participant's button press. The estimated average reaction time was subtracted from the time of button presses to model the actual timing of rivalry-switch and replay-switch events. Six head-motion parameters were also included in the GLM model as regressors of no interest to model and eliminate any noise on EPI images due to motion during the scanning.

### Dynamic causal modeling analysis

Dynamic causal modeling (Friston et al., 2003) was performed using DCM12 in SPM12 (Wellcome Trust Centre for Neuroimaging). DCM analysis for fMRI data aims to infer influence of neural activity by describing changes of BOLD signal as a function of experimental condition.

DCM models describe changes in connectivity as follows:

$$\frac{dx\left(t\right)}{dt}=\left(A+\sum_{j=1}^{m}u^{j}B^{\left(j\right)}\right)x\left(t\right)+C_{u}$$

Here, *x*(*t*) represents brain activations in ROIs and *t* expresses time, so the equation describes time-dependent changes in the dynamics of brain activation. The right side of the equation shows that the rate of change in brain activity in an ROI can be represented by the combination of brain dynamics of other regions and experimental conditions: *A* represents the endogenous connectivity matrix (A-matrix) and thus represents context-independent connectivity between regions. Therefore in the present experiment, the values in the A-matrix were the same across conditions. Contextual variables (e.g. experimental conditions) are denoted by the vector *u* and the matrix *B* represents the modulations on endogenous connectivity (B-matrix). C represents driving input (C-matrix) and models the effect of experimental condition (*u*) on the brain dynamics in the ROI. The values of the B-matrix and C-matrix are expected to vary depending on the experimental conditions. A recent study employing electrophysiological recording confirmed that DCM analysis can locate the source of neural activations better than similar analytic tools such functional connectivity and Granger causality, implying that DCM parameters can characterize neural dynamics in a biologically and functionally meaningful way (David et al., 2008).

In our study, we were particularly interested in investigating B-matrix parameters during rivalry-switch and replay-switch events and relate them to individual differences in behavioral data. We hypothesized that r-aSPL, r-pSPL, and r-V5 constitute a three-layer hierarchical model with reciprocal interactions between areas during spontaneous perceptual transitions (Kanai et al., 2011) and tested this hypothesis by estimating these coupling parameters (B-matrix) in DCM. The three ROIs were selected based on univariate fMRI results: r-aSPL, r-pSPL, and r-V5. ROIs for DCM analysis were defined by the following procedure. First, peak voxel coordinates were found for each ROI based on anatomically defined ROIs: 10 mm radius sphere centered (*x*, *y*, *z*) = (36, − 45, 51) for r-aSPL (Carmel et al., 2010), 10 mm radius sphere centered (38, − 64, 32) for r-pSPL (Kanai et al., 2011), and 10 mm radius sphere centered (44, − 67, 0) for V5/MT (Dumoulin et al., 2000; Mars et al., 2011). Then the 10 mm-sphere masks centered on the peak voxels were created with PickAtlas (Maldjian et al., 2003) and applied to group level fMRI results (Rivalry-switch > Replay-switch contrast, thresholded at *p <* 0.001, uncorrected) to create DCM ROIs (See Fig. 3). Averaged BOLD signals in each region were extracted using the *Volume of Interest* module in SPM8 and used for DCM analysis.

DCM analysis was performed in two steps. First, we explored the optimal model structure that best described neuronal responses using Bayesian model selection (DCM model selection). Four conditions (fixation, visual stimulation, rivalry-switch, and replay-switch) in the GLM model were included in DCM models. A previous functional connectivity study has shown that the posterior part of parietal lobule is specifically coupled to V5/MT in the resting state (Mars et al., 2011) and therefore our DCM models specifically posited endogenous connectivity between r-pSPL and r-V5 but not r-aSPL and r-V5. It is recommended to utilize such prior knowledge about brain connectivity when defining DCM model space (Stephan et al., 2010) and both monkey electrophysiology (Vincent et al., 2007) and imaging (Greicius et al., 2009) studies suggest that functional connectivity reflects anatomical connectivity. Direct input to r-V5 (C-matrix) was explicitly modeled during visual stimulation, rivalry-switch, and replay-switch events but not during the fixation period. Human area V5/MT is involved in visual motion processing (Tootell et al., 1995) and a previous structure-from-motion study also detected activation in V5/MT associated with subjective perceptual switches (Freeman et al., 2012), suggesting V5/MT is involved in perceptual switches even when the stimulus remains unchanged (albeit with a repetitively fluctuating retinal input due to the sinusoidal motion of individual dots). In addition, theoretical work proposes that adaptation of neurons in visual cortex may be the driving force for perceptual switches in bistable perception (e.g. Dayan, 1998). These studies indicate that direct input to r-V5 should be included in our modeling of perceptual switches. We therefore modeled modulatory effects on four connections between ROIs (from r-aSPL to r-pSPL, from r-pSPL to r-aSPL, from r-pSPL to r-V5, and from r-V5 to r-pSPL—see Fig. 3 for ROI positions) and two driving inputs (r-aSPL and r-pSPL) in rivalry-switch and replay-switch events and therefore the total number of tested models was 64 (6 dimensions, 2^4^ for B-matrix × 2^2^ for C-matrix). We then divided all the models into four groups based on modulatory parameters: no modulation, top-down, bottom-up, and bidirectional (See Fig. 4). The exceedance probability of each family and model was computed with random-effect assumptions using a Bayesian approach and the best-fit model in the winning group was selected as the winning model (Penny et al., 2004, 2010). The exceedance probability represents the probability that a model or model family is more likely than the other models or families. More detailed description can be found elsewhere (Penny et al., 2010; Stephan et al., 2009). Note that we assumed that the optimal model structure was common between rivalry-switch and replay-switch condition and this assumption enabled us to handle DCM parameters quantitatively across the two conditions and relate them to behavioral performance.

### Individual difference analysis

To investigate whether the DCM parameters could explain the variations in individual behavioral differences between participants, a multiple regression analysis was performed using SPSS software (International Business Machines Corporation, New York). We tested the hypothesis that the variability in network dynamics is related to inter-individual variability in behavior. We asked whether the difference in the strength of modulatory effect (B-parameters) for rivalry-switch and replay-switch conditions was predictive of the behavioral variability across participants. Thus, we chose the difference between B-parameters of the winning model (i.e. rivalry − replay) as regressors and tested if those regressors could predict an individual's mean dominance duration.

To ensure a good fit to the regression model and remove outlier effects, three participants whose Cook's distance was larger than one were eliminated from the analysis. Cook's distance is a measure to detect influential data points in regression analysis and used for detecting outliers (Cook, 1977). We report *R*^2^ and corrected *R*^2^ value (adjusted for degree of freedom to account for number of repressors).

## Results

### Behavioral data analysis

The average perceptual dominance duration across the two fluctuating bistable percepts was 4.50 s (SD: 0.99). In the replay condition, mean dominance duration was 4.48 s (SD: 1.00) and there was no significant difference in mean dominance duration of the two conditions (*t*(17) = 0.69, *p* = 0.50, n.s.), suggesting that participants successfully reported replay based on the depth information added to the stimuli.

### GLM analysis

**Fig. 2:**
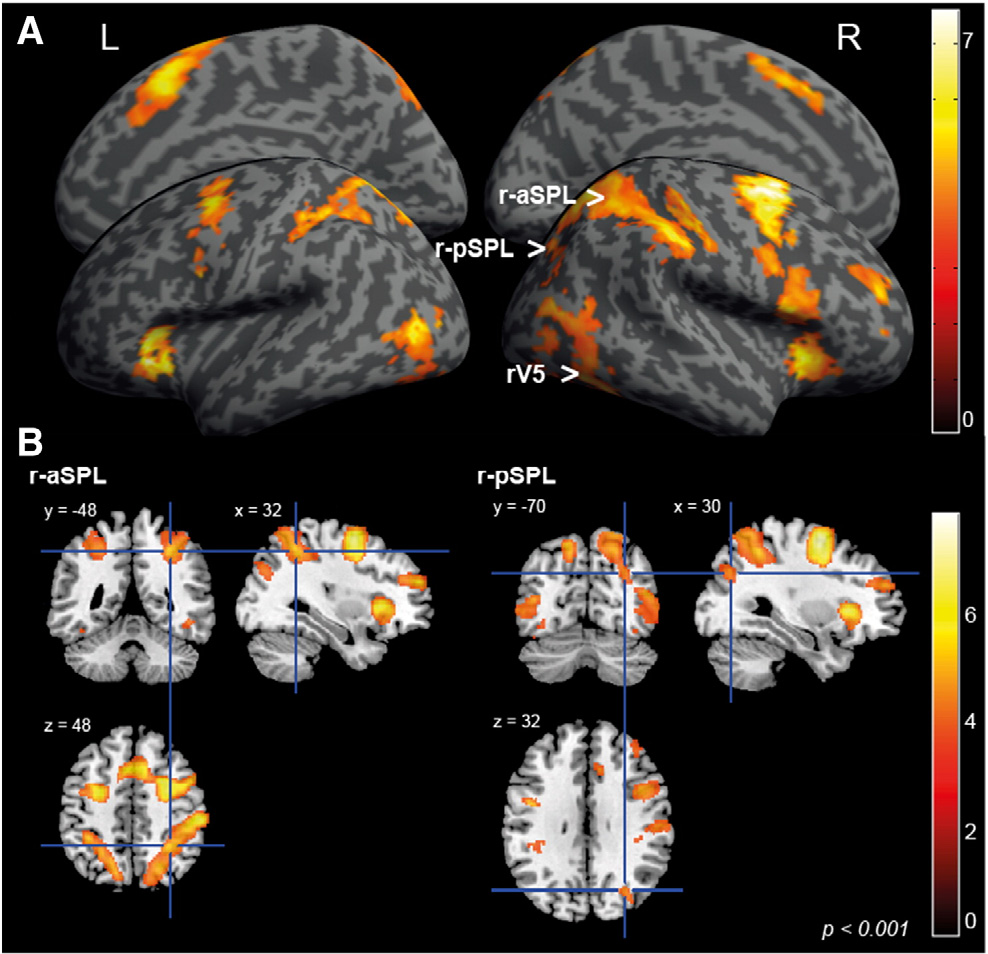
Brain activation evoked by perceptual switches (second level analysis; *p* < 0.001, uncorrected). (A) Activation associated with perceptual transitions in the rivalry condition (rivalry-switch) comparing to the replay condition (replay-switch) is shown in this figure. The color bar indicates the T-value of the GLM activation map which is overlaid on a MNI template artificially ‘inflated’ using SPM8. (B) The figure shows the peak voxel coordinates of r-aSPL (the left panel, (32, − 48, 48), *p* < 0.01, corrected for small-volume) and r-pSPL (the right panel, (30, − 70, 32), *p* < 0.01, corrected for small-volume). The color bar indicates T-value of the GLM activation map overlaid on an MNI anatomical template brain using MRICron (http://www.mccauslandcenter.sc.edu/mricro/mricron/).

To investigate which brain regions showed activation associated with perceptual transitions, we constructed a general linear model (GLM) that included stimulus presentation, fixation, spontaneous perceptual change (rivalry-switch), and stimulus-driven perceptual change (replay-switch) as regressors. Fig. 2A shows the brain activations correlated with the rivalry-switch regressor compared to replay-switch. Given our prior hypothesis (Carmel et al., 2010; Kanai et al., 2010; Lumer et al., 1998) we used small volume correction (SVC) and validated that the activation evoked during rivalry-switch in r-aSPL (sphere radius = 10 mm, sphere center, (36, − 45, 51); peak voxel, (32, − 48. 48), *t*(17) = 5.38, *p* = 0.002, *p* < 0.01, corrected for small-volume) and r-pSPL (sphere radius = 10 mm, sphere center, (38, − 64. 32); peak voxel, (30, − 70. 32), *t*(17) = 4.47, *p* = 0.01, *p* < 0.01, corrected) (see Fig. 2B). Moreover, motion-sensitive visual area V5/MT in the right hemisphere also showed greater activation associated with rivalry versus replay switches (sphere radius = 10 mm, sphere center, (44, − 67, 0); peak voxel, (48, − 62, − 8), *t*(17) = 4.82, *p* = 0.005, *p* < 0.01, corrected). We performed additional GLM analysis with regressors accounting for any differences in the stimuli between rivalry condition and replay condition (Text S1) and found a similar result (Fig. S1), suggesting r-aSPL and r-pSPL activation was not merely due to difference in stimulus condition (presence of binocular disparity).

In addition, we also observed activation evoked by spontaneous perceptual switches in frontal cortex, visual cortex, insula, and middle frontal gyrus (see Fig. 2A and Table 1) as reported in previous studies of bistable perception (Kleinschmidt et al., 1998; Knapen et al., 2011; Sterzer and Kleinschmidt, 2007; Zaretskaya et al., 2010).

### Dynamic causal modeling analysis

**Fig. 3:**
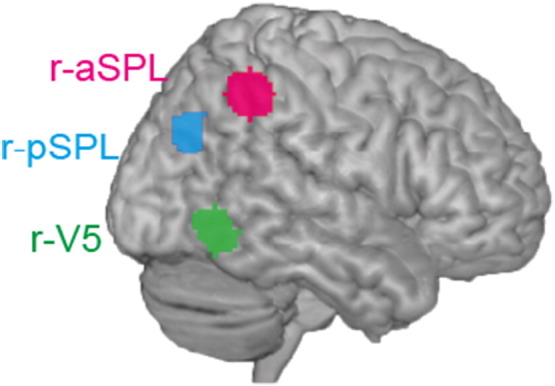
Regions of interest for DCM analysis. r-aSPL (red), r-pSPL (blue), and r-V5 (green) were identified based on anatomical coordinate and univariate analysis (rivalry-switch > replay-switch; see Method and Result for details). We confirmed that r-aSPL and r-pSPL ROIs were consistent in location with previous reports (Kanai et al., 2011) (shown as magenta and cyan in the figure respectively; see main text for details).

Having established that activity in r-aSPL and r-pSPL was associated with perceptual switches, DCM was performed to characterize the dynamic coupling between three ROIs (Fig. 3). r-aSPL (435 voxels), r-pSPL (152 voxels), and r-V5 (356 voxels) were selected based on the GLM results (rivalry-switch > replay-switch) as described in the previous section.

**Fig. 4:**
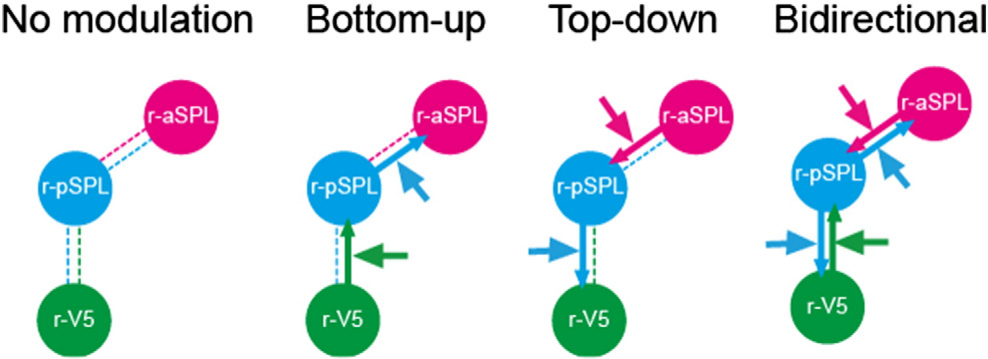
DCM model families for model comparison are illustrated. 64 models were divided into four model families (No modulation, 4 models; Bottom-up, 12 models; Top-down, 12 models; Bidirectional, 36 models) according to modulatory effect. Figure describes modulatory effect (B-matrix) and models with different driving inputs (four patterns) were included in the family.

**Fig. 5:**
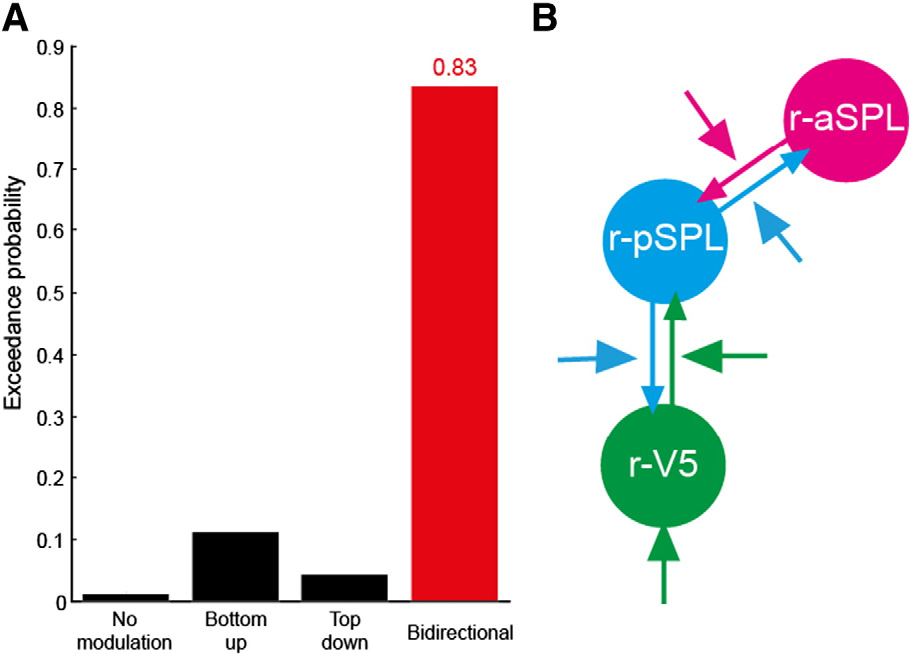
Results of DCM analysis. (A) DCM family-level model comparison result is shown in this figure. Random-effect Bayesian comparison indicates that bidirectional model family (i.e. models containing bottom-up and top-down modulatory effects) was the best among the four families. (B)Winning model contains four modulatory inputs to all connections and driving input to r-V5 (exceedance probability for the winning model was 0.44 among 64 models).

To find the optimal DCM model structure that described the interaction between these regions associated with each experimental condition, we used family-level Bayesian model selection. 64 models (combination of all possible models) were divided into four families based on their underlying B-matrix: no modulation, bottom-up, top-down, and bidirectional (Fig. 4). We found that the exceedance probability was largest for the bidirectional family of the models (Fig. 5A; the exceedence probability of the winning model family was 0.83). We confirmed the bidirectional model family was the best among the four families using different ROI selection approach (Fig. S2A) and GLM (Fig. S2B).

The winning model in the bidirectional family was the model described in Fig. 5B (the exceedance probability of the winning model was 0.44 among all 64 models). There were four modulatory effects in the winning model: (r-aSPL to r-pSPL), (r-pSPL to r-aSPL), (r-pSPL to r-V5), and (r-V5 to r-pSPL). In addition, r-V5 received driving input during perceptual transitions. In DCM, the strength of parameters characterizes how the rate of activation changes in a region is affected by activation in a given connected region. Here, positive values indicate that increasing activation in a region facilitates the rate of change in the connected region whereas negative values mean increasing activation in a region suppresses the rate of change in the connected region (Seghier et al., 2010).

**Fig. 6:**
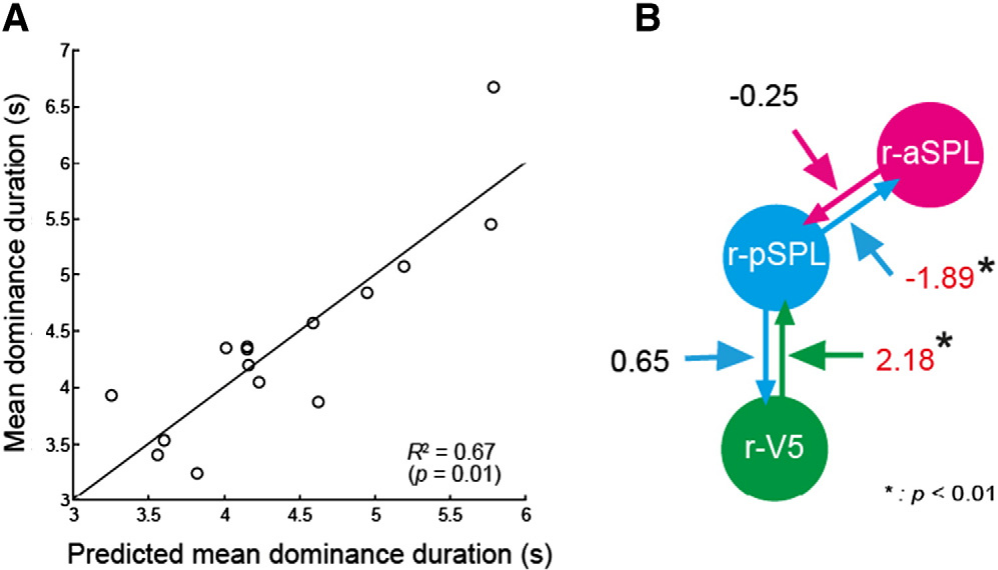
Result of multiple regression analysis. (A) Multiple regression analysis showed that a combination of four B-parameters could predict an individual's mean dominance duration. The *R*^2^ value given in the figure is adjusted *R*^2^. (B) Two bottom-up modulatory parameters (r-pSPL to r-aSPL and r-V5 to r-pSPL) were the significant predictors for individual mean dominance duration (r-pSPL to r-aSPL, *p* = 0.003, *p* < 0.01; r-V5 to r-pSPL, *p* = 0.002, *p* < 0.01). Values besides the arrows indicate *β* (standardized coefficients) of each predictor in the full-model.

Finally, multiple regression analysis was performed to explore if the parameters in the winning DCM model could predict variation in the individual behavioral data (mean percept dominance duration). Differences in four DCM B-parameter values between the two switch conditions were entered into a multiple linear regression model as predictors. The model successfully predicted the individual mean dominance duration (*R*^2^ = 0.77 (adjusted *R*^2^ = 0.67), *F*(4, 10) = 8.18, *p* = 0.003; see Fig. 6A). Specifically, two bottom-up B-parameters were significantly correlated with mean dominance duration in the full model (*β* = − 1.896, *t*(10) = − 3.919, *p* = 0.003, *p* < 0.01 for r-pSPL to r-aSPL; *β* = 2.18, *t*(10) = 4.30, *p* = 0.002, *p* < 0.01 for r-V5 to r-pSPL; see Fig. 6B): suppressive modulation from r-pSPL to r-aSPL and facilitative modulation from r-V5 to r-pSPL were associated with a longer dominance duration. We did not observe such trends in the two top-down B-parameters ((*β* = − 0.25, *t*(10) = − 1.28, *p* = 0.23, *n.s.* for r-aSPL to r-pSPL; *β* = 0.65, *t*(10) = 2.05, *p* = 0.07, *n.s.* for to r-pSPL to r-V5).

## Discussion

Here, we investigated how two focal areas of parietal cortex and the motion-sensitive area V5/MT of the human brain interacted with each other during visual perceptual switches in bistable perception. Using DCM analysis, we formally characterized reciprocal modulatory interactions between these brain areas which were designated by our prior hypothesis (Kanai et al., 2011). Furthermore, we found that the strength of bottom-up modulations accounted for inter-individual variability in percept dominance duration.

We first replicated the previously described functional association between activity in human parietal regions and perceptual switches. Lumer et al. (1998) showed that higher BOLD responses in the superior parietal lobule (SPL) are observed during perceptual switches in binocular rivalry. Kanai et al. (2010, 2011) showed that cortical gray matter volume and thickness of r-aSPL and r-pSPL correlate with perceptual switch rate for structure from motion (SFM). In addition, modulation of subjective perception by application of TMS to these areas confirmed a causal role for these regions in bistable perception (Carmel et al., 2010; Kanai et al., 2010, 2011). Despite this compelling collection of evidence for the role of right human SPL in fluctuations of subjective awareness, the functional interplay between these parietal subregions and lower visual areas has not previously been shown.

It has been suggested that perceptual switches are caused by continuous cortical interactions between fronto-parietal regions and sensory regions rather than just “bottom-up (feed-forward)” or “top-down (feedback)” neural communication (Sterzer et al., 2009). Previous TMS and fMRI studies (Kanai et al., 2011; Zaretskaya et al., 2010) pointed to a role for connectivity between a number of parietal and visual brain areas in the human brain in bistable perception. Multiple brain regions, including visual cortex and fronto-parietal regions, show activation when perceptual switches occur and this has been replicated several times; see Rees (2007). In addition, a recent fMRI study (Wang et al., 2013) suggested that changes in functional connectivity (Friston et al., 2013) between multiple brain regions is enhanced during a bistable perception task further supporting the role of connectivity changes in bistable perception. Despite this wide range of previous findings, whether fronto-parietal activation associated with perceptual switches directly contributes to conscious perception is contested: for example, activations of fronto-parietal regions could reflect top-down information processes such as selective attention (Sterzer et al., 2009). Alternatively, a recent study proposed that activations observed in the fronto-parietal regions are due to ambiguity in visual information rather than a driving force of perceptual switches (Knapen et al., 2011). Yet another more recent study has attributed this brain activity to introspection and report of perceptual states (Frassle et al., 2014) rather than a change in the subjective content of consciousness. These results cast doubt on the involvement of fronto-parietal areas in perceptual alternation.

To address this issue directly, we used DCM analysis to identify the dynamics of network level interactions between parietal and motion sensitive visual areas during perception of bistable structure from motion. The winning model (Fig. 5B) comprised four bidirectional connections in which r-V5 is both a driving force as well as modulated by perceptual switches. This structure indicates that sensory input to r-V5 propagates to higher brain areas (r-aSPL and r-pSPL); and r-V5 and r-pSPL both receive feedback modulation, suggesting that perceptual switches are induced as a result of bidirectional modulation between fronto-parietal and sensory areas. Furthermore, we found that the variation in bottom-up modulatory parameters (B-parameters) between the rivalry and replay conditions could predict the individual participant's mean dominance duration. Although further studies will be required to understand the precise nature of the biological mechanisms that account for the difference between the two bottom-up modulations, the correlation between DCM parameters and mean dominance duration is evidence for the involvement of the two parietal regions in perceptual switches.

How does the bidirectional interaction described here give rise to changes in perceptual states? The predictive coding theory of brain function (Clark, 2013; Helmholtz, 1910; Rao and Ballard, 1999) offers a framework to answer this question. This theory proposes that the brain seeks to infer the causes in the external world that give rise to the signals gathered through sensory organs. Based on these inferences, the brain constructs expectations or predictions about subsequently forthcoming sensory input which are then iteratively updated by comparing the expectations with the observation and computing the “prediction error” (Hohwy et al., 2008). The neuronal correlates of such iterative prediction and comparison processes have been documented in several brain regions when participants engage in visual tasks (Muckli et al., 2005; Murray et al., 2002; Summerfield et al., 2006). Recent theoretical (Hohwy et al., 2008) and empirical work (Denison et al., 2011) have also suggested that predictive coding theory could account for perceptual alternation in bistable perception.

Kanai et al. (2011) employed this framework to propose a connectivity hypothesis consisting of r-aSPL, r-pSPL and visual cortex that might account for bistable perception. Based on the observation that impairing r-pSPL and r-aSPL function by TMS prolongs and shortens, respectively, the mean dominance duration in bistable structure from motion perception, they proposed that r-aSPL generates a prediction about the causes of sensory evidence (i.e. structure of the environment) and r-pSPL computes the prediction error between that expectation and the sensory evidence it receives from the visual cortex. Our results showed that the connectivity structure of our winning DCM model is consistent with the connectivity hypothesis proposed by Kanai et al. (2011).

Taken together, we speculate that the bottom-up modulation from r-pSPL to r-aSPL (and from V5/MT to r-pSPL) corresponds to a hierarchical process of “explaining away” which may serve to balance out the difference between prediction (represented in r-aSPL) and sensory information (represented in r-V5). In this view, smaller prediction errors (i.e. less bottom-up modulation from r-pSPL to r-aSPL and from V5/MT to r-pSPL) would lead to stabilized perception (longer mean dominance duration) as demonstrated in our findings.

Another recent fMRI study drew a rather different conclusion regarding regional interaction in perceptual switches. Weilnhammer et al. (2013) explored perceptual alternations associated with a rotating Lissajous figure and demonstrated that a DCM model with top-down modulation (but no bottom-up modulation) from the right inferior frontal gyrus (rIFG) to the right V5/MT could account for the neural dynamics of spontaneous perceptual switches. The difference between the present and previous studies may be associated with differences in paradigm, but may also come from ROI selection process. In our study, we included both anterior SPL and posterior SPL in DCM models separately based on the hypothesis from previous study (Kanai et al., 2011) and did not include rIFG. DCM analysis should be performed using anatomical or functionally connected regions (Stephan et al., 2010) and parietal regions and V5/MT are indeed anatomically and functionally connected (Mars et al., 2011). Most importantly, we found the strength of two bottom-up connections predicted individual mean dominance duration and this implies involvement of bottom-up connectivity in defining the timing of perceptual alternation, at least for our structure-from-motion stimulus.

In summary, we found that activity in two focal regions of parietal cortex plus motion-sensitive visual cortex influenced each other during bistable perceptual switches; and the strength and direction of modulation of connectivity between regions predicts individual mean percept dominance duration. Our results are consistent with a predictive-coding theory of bistable perception and contribute to clarifying the dynamics of a functional network in the brain that contributes emergence of conscious perception.

## Figures and Tables

**Table 1:** ROI table for Rivalry > Replay contrast. Regions are labeled with AAL—Anatomical Automatic Labeling tool (Tzourio-Mazoyer et al., 2002).

AAL label	*t*(17)	*p* (uncorrected)	Peak coordinate			Number of voxels
			*x*	*y*	*z*	
Frontal_Sup_R	8.65	< 0.001	18	8	66	5187
Insula_L	8.24	< 0.001	− 34	22	2	718
Occipital_Mid_L	6.71	< 0.001	− 44	− 78	10	775
Postcentral_R	6.54	< 0.001	56	− 26	48	4888
Frontal_Mid_L	6.31	< 0.001	− 26	− 4	50	820
Parietal_Sup_L	6.18	< 0.001	− 18	− 62	52	1559
Frontal_Mid_R	5.60	< 0.001	32	52	22	544
Occipital_Inf_R	4.01	< 0.001	38	− 78	− 14	62
Temporal_Inf_L	4.01	< 0.001	− 42	− 48	− 16	8
Thalamus_R	3.71	0.001	8	− 14	0	3
