# Supplementary material for: Brain activity dynamics in human parietal regions during spontaneous switches in bistable perception
Source: Neuroimage. 2015 Feb 15;107:190–7. doi: 10.1016/j.neuroimage.2014.12.018 (PMC4306523; doi:10.1016/j.neuroimage.2014.12.018)
Supplement: Supplementary file 1 — Supplementary material [file mmc1.docx]

Supplementary Material

NIMG-14-1306R1

Brain activity dynamics in human parietal regions during spontaneous switches in bistable perception

Fukuda Megumi ^a^, Bahador Bahrami ^a^, Ryota Kanai ^b^, and Geraint Rees ^a, c^

1. Institute of Cognitive Neuroscience, University College London, 17 Queen Square, London, WC1N 3AR, United Kingdom
2. Sackler Center for Consciousness Science, School of Psychology, University of Sussex, Pevensey 1, Brighton, BN1 9QH, United Kingdom
3. Wellcome Trust Centre for Neuroimaging, University College London, 12 Queen Square, London WC1N 3BG, United Kingdom

# Supplementary Text

## Additional GLM

We also analyzed our fMRI data (see Methods) using an additional GLM (alternative GLM) to confirm that activation in r-aSPL and r-pSPL associated with the comparison of rivalry-switch and replay-switch (Figure 2) did not merely reflect minor differences in stimuli between the conditions (i.e. presence of binocular disparity). We employed the same preprocessing procedure as the original GLM (see Method section in the main text) but the design matrix of the new GLM model now comprised five main regressors: fixation, spontaneous perceptual switch (rivalry-switch), stimulus-driven perceptual switch (replay-switch), stable percept in rivalry block (rivalry-stable, i.e. stimulus presentation without perceptual switch in rivalry block), and stable percept in replay block (replay-stable, stimulus presentation without perceptual switch in replay block). Fixation periods, rivalry-stable, replay-stable were modeled using a box-car function, which represented the onset and duration of fixation period and stable percept. Rivalry-switch and replay-switch were modeled with an impulse function; the estimated average reaction time was subtracted from the time of button presses to model the actual timing of rivalry-switch and replay-switch events (See ‘MRI Data Processing’ section in the main text). Six head motion parameters derived from the preprocessing were also included in this GLM to regress out nuisance signal due to head movement.

This alternative GLM showed results (Figure S1) similar to the original GLM; r-aSPL and r-pSPL showed greater activation in rivalry switch comparing to replay-switch (r-aSPL peak voxel, (34, -52, 50), *t*(17) = 3.75 , *p* = 0.001, uncorrected; r-pSPL peak voxel, (30, -70. 32), *t*(17) = 3.11, *p* = 0.003, uncorrected).

## Additional DCM analysis

### DCM with individual ROIs

To validate the robustness of our DCM result, additional DCM analyses were performed and we demonstrated that the winning model family remained the same despite different ways of defining ROIs (Figure S2A).

First, we tested a different DCM ROI selection approach. In the main text, we report the DCM result using the same ROI mask for all participants. Previous network connectivity studies show that inappropriate signal in ROIs may lead to failure to detect network connectivity (Smith et al., 2011). We accounted for individual differences in the ROI peak location across participants by instead selecting voxels for ROIs using the individual first level GLM activation map. DCM ROIs were created as follows: 10mm spherical ROI masks centered on the original second level GLM (See Figure 2 for activation map) were created and applied to each individual’s GLM activation map for the contrast Rivalry > Replay (see Table S1 for individual peak coordinates). Voxels that exceeded a liberal threshold of P < 0.3 (uncorrected) within that sphere were used collectively as ROIs for DCM analysis. Note that two participants out of eighteen were excluded from this analysis due to the absence of any activated voxels at this threshold inside the 10mm ROI spheres.

Bayesian family-level comparison revealed that the bidirectional model family was the best among the four families (exceedence probability 0.92).

### DCM with the alternative GLM

We also performed family-level DCM model comparison with the alternative GLM to explore if difference in regressors would affect DCM family level inference (Figure S2B). We defined DCM ROIs with similar procedure used for the main DCM analysis: 10mm-sphere masks centered on the group level peak voxel coordinates ((34, -52, 50) for r-aSPL, (30, -70, 32) for r-pSPL, and (44, -74, 6) for r-V5) were applied to group level fMRI results (Rivalry-switch > Replay-switch contrast, thresholded at *p* *<* 0.008, uncorrected) and used as DCM ROIs.

Again, we found bidirectional model family was the most likely DCM model family among the four families (exceedance probability was 0.98).

Table S1 Individual peak coordinate of r-aSPL, r-pSPL, and r-V5 within DCM ROIs (See Figure S2A).

|  |  | r-aSPL | | |  | r-pSPL | | |  | r-V5 | | |
| --- | --- | --- | --- | --- | --- | --- | --- | --- | --- | --- | --- | --- |
|  |  | *X* | *y* | *z* |  | *x* | *y* | *z* |  | *x* | *y* | *z* |
| Subject 1 |  | 26 | -52 | 52 |  | 30 | -62 | 38 |  | 42 | -58 | -14 |
| Subject 2 |  | 38 | -48 | 54 |  | 30 | -74 | 30 |  | 48 | -58 | -14 |
| Subject 3 |  | 34 | -52 | 48 |  | 32 | -64 | 34 |  | 52 | -66 | 0 |
| Subject 4 |  | 36 | -42 | 54 |  | 30 | -78 | 38 |  | 52 | -62 | 0 |
| Subject 5 |  | 32 | -40 | 42 |  | 38 | -70 | 38 |  | 54 | -58 | -6 |
| Subject 6 |  | 36 | -48 | 42 |  | 28 | -66 | 30 |  | 42 | -58 | -10 |
| Subject 7 |  | 36 | -50 | 54 |  | 24 | -66 | 38 |  | 48 | -66 | 0 |
| Subject 8 |  | 38 | -42 | 50 |  | 32 | -76 | 28 |  | 48 | -66 | -6 |
| Subject 9 |  | 28 | -54 | 46 |  | 34 | -68 | 36 |  | 54 | -58 | -14 |
| Subject 10 |  | 38 | -54 | 52 |  | 34 | -64 | 26 |  | 52 | -66 | -10 |
| Subject 11 |  | 36 | -42 | 50 |  | 32 | -64 | 36 |  | 50 | -58 | 0 |
| Subject 12 |  | 36 | -40 | 52 |  | 28 | -72 | 24 |  | 44 | -64 | -16 |
| Subject 13 |  | 28 | -40 | 44 |  | 26 | -72 | 36 |  | 48 | -70 | -2 |
| Subject 14 |  | 32 | -42 | 40 |  | 26 | -76 | 36 |  | 48 | -68 | 0 |
| Subject 15 |  | 36 | -46 | 44 |  | 30 | -62 | 38 |  | 42 | -58 | -6 |
| Subject 16 |  | 40 | -44 | 46 |  | 26 | -62 | 36 |  | 54 | -56 | -6 |


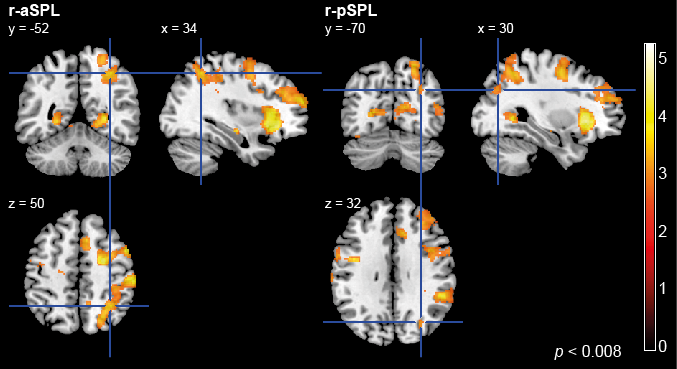


Figure S1 GLM validation analysis (second level analysis; *p* < 0.008, uncorrected). We found activation in r-aSPL and r-pSPL (r-aSPL peak voxel, (34, -52, 50), *t*(17) = 3.75 , *p* = 0.001, uncorrected; r-pSPL peak voxel, (30, -70. 32), *t*(17) = 3.11, *p* = 0.003, uncorrected).


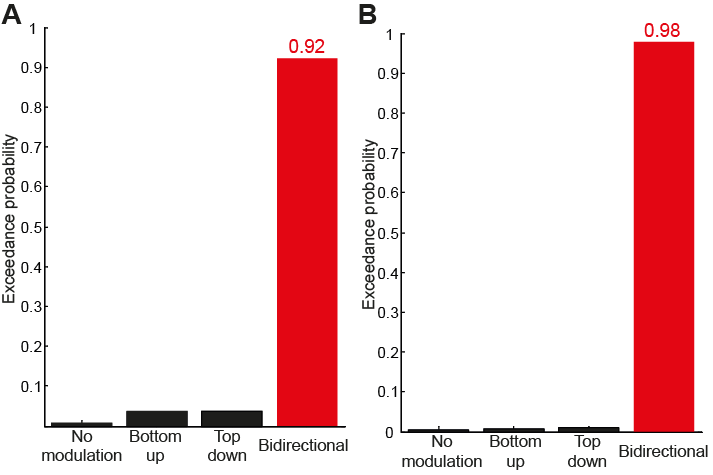


Figure S2 DCM validation analyses.

(A) We also performed another DCM analysis with individual-based ROI selection approach. Based on the original GLM (See Figure 2 for activation map), three ROI masks were created based on individual GLM activation map (See Table S1 for peak coordinates) and DCM family-model comparison revealed bidirectional model family (Figure 4) was the winning model family. (B) To address if difference in GLM would affect DCM result, we performed DCM analysis with the alternative GLM (Figure S1).. Again that bidirectional model family (Figure 4) was the best among four families with the alternative GLM.

# Reference

Smith, S.M., Miller, K.L., Salimi-Khorshidi, G., Webster, M., Beckmann, C.F., Nichols, T.E., Ramsey, J.D., Woolrich, M.W., 2011. Network modelling methods for FMRI. Neuroimage 54, 875-891.
